# Comparative Immunomodulatory Activity of *Nigella sativa* L. Preparations on Proinflammatory Mediators: A Focus on Asthma

**DOI:** 10.3389/fphar.2018.01075

**Published:** 2018-10-02

**Authors:** Abdulrahman E. Koshak, Nizar M. Yousif, Bernd L. Fiebich, Emad A. Koshak, Michael Heinrich

**Affiliations:** ^1^Department of Natural Products and Alternative Medicine, Faculty of Pharmacy, King Abdulaziz University, Jeddah, Saudi Arabia; ^2^Research Group Pharmacognosy and Phytotherapy, UCL School of Pharmacy, London, United Kingdom; ^3^Faculty of Biology, University of Freiburg, Freiburg, Germany; ^4^VivaCell Biotechnology, Denzlingen, Germany; ^5^Allergy and Clinical Immunology Division, Department of Internal Medicine, Faculty of Medicine, King Abdulaziz University, Jeddah, Saudi Arabia

**Keywords:** NS, black Seed, anti-inflammatory, immunomodulatory, cytokines, *in vitro*

## Abstract

**Introduction:** A range of traditional and commercial preparations of NS is frequently used in the treatment of several inflammatory diseases. Often, these preparations have poor preclinical characterization that may lead to variable pharmacological effects.

**Objective:** To assess the *in vitro* effects of different chemically defined preparations of NS on some asthma-related mediators of inflammation.

**Methods:** Different NS preparations were obtained by either seed extraction with a spectrum of solvents ranging from lipophilic to hydrophilic, or commercial products were collected. The TQ concentration of NS was analyzed by HPLC. Immunomodulatory activity was assessed by the release of mediators (IL-2, IL-6, PGE_2_) in primary human T-lymphocytes, monocytes, and A549 human lung epithelial cells.

**Results:** Ten distinct NS preparations showed variability in TQ concentration, being highest in the oily preparations extract-7 (2.4% w/w), followed by extract-10 (0.7%w/w). Similarly, the release of mediators was varied, being greatest in extract-7 and 10 via significantly (<0.05) suppressing IL-2, IL-6, and PGE_2_ in T-lymphocytes as well as IL-6 and PGE_2_ in monocytes. Also, PGE_2_ release in A549 cells was significantly enhanced by both extracts.

**Conclusion:** The TQ concentration and *in vitro* activity were variable among the different NS preparations. TQ-rich oily NS preparations produced potent favorable immunomodulation in asthma inflammation and can be used in future studies.

## Introduction

The seeds of the plant NS family Ranunculaceae are commonly used as a spice known as black cumin. It has traditional medical applications and is considered to be a characteristic herbal medicine for diverse diseases in Unani, Indian, Arabic, and Prophetic traditional medical traditions (Ahmad et al., 2013). Avicenna reported its benefit for shortness of breath and for stopping phlegm (Avicenna, 1593). Dioscorides reported its medical use for cleaning rough skin and psoriasis (Osbaldeston, 2000). Hippocrates used it for treating hepatic and digestive conditions (Salama, 2010). Imam Ibn Qayyim Al-Jawziyya (1292–1350 AD), the author of the Prophetic Medicine, reported that NS alleviate gasping and hard breathing (Abdullah, 2003). In Arabia, NS is a traditional remedy for asthma, cough, stomach ache, abdominal pain, colic, general fatigue, rheumatism, and skin diseases (Lebling and Pepperdine, 2006).

NS seeds contain fixed oil (24.76 to 40.35%), volatile oil (0.5 to 1.6%), quinones, alkaloids, saponins, and other compounds in trace amounts (Liu et al., 2011; Botnick et al., 2012; Ahmad et al., 2013). The compounds responsible for NS’s activities are still not well-established. It is likely that several compounds are responsible or co-responsible for NS activity and that one must consider a specific extract as the active ingredient rather than a single compound. TQ is the main component of NS’s volatile oil and based on several studies it is considered to be a key active ingredient (Ahmad et al., 2013). Other compounds such as dithymoquinone, thymohydroquinone, thymol, carvacrol, and α-hederin were also reported to produce relevant pharmacological effects in preclinical models (Marsik et al., 2005; Landa et al., 2009b; Fallahi et al., 2016). As obvious with other herbal medicines, the extraction method influences the chemical composition and, consequently, the pharmacological activity of a specific NS preparation.

The anti-inflammatory/immunomodulatory activity of NS was recently reviewed in asthma. NS extracts and/or its active constituents (including TQ, nigellone, and alpha-hederin) showed anti-histaminic, anti-eosinophilic, anti-leukotrienes, anti-immunoglobulin and reduced proinflammatory cytokines (interleukins-2, 4, 5, 6, 12, and 13) in *in vitro/in vivo* models (Koshak et al., 2017a).

Asthma is a common respiratory disease affecting about 334 million people worldwide and has become a global health priority (Global Asthma Network, 2014). It is a chronic airway inflammatory disease recognized by a history of respiratory symptoms such as wheezing, shortness of breath, chest tightness, and cough accompanied by lung hyper-responsiveness and inflammation (Global Initiative for Asthma, 2017). The pathology of asthma is initiated by multiple interactions between inflammatory cells and mediators. The most important cells involved in asthma-related inflammation are mast cells, eosinophils, T lymphocytes, neutrophils, and epithelial cells (Koda-Kimble, 2009). Typically in asthma, cytokines released by Type 2 T helper (Th2) cells including interleukin-4 (IL-4), interleukin-5 (IL-5), and interleukin-13 (IL-13) as well as cytokines released by Type 1 T helper (Th1) cells such as interferon-gamma are considered to be the main cytokines triggered in asthma (Ngoc et al., 2005).

Nevertheless, other proinflammatory mediators are also involved in asthma-related inflammation. For instance, interleukin-2 (IL-2) was found to be increased in the broncho-alveolar lavage fluid (BALF) of patients with asthma (Virchow et al., 1996). IL-2 may increase the production of IL-5 and attract eosinophils (van Haelst Pisani et al., 1991; Kuraoka et al., 2004; Davoine and Lacy, 2014). Moreover, patients treated with inhaled IL-2 had asthma-like signs and symptoms (Loppow et al., 2007). Interleukin-6 (IL-6) is a major pro-inflammatory mediator and a relevant target for several diseases (Hirano, 1998). IL-6 expression was increased in bronchial epithelial cells of patients with asthma (Mattoli et al., 1992). In patients with asthma, IL-6 was found at a high level in serum as well as in BALF (Yokoyama et al., 1995; Tillie-Leblond et al., 1999). IL-6 was elevated in stimulated sputum of allergic asthma patients (Neveu et al., 2010). Elevated serum levels of IL-6 were found in 54% of 170 adult asthmatics (Ilmarinen et al., 2016).

Additionally, prostaglandin E_2_ (PGE_2_) is commonly assumed to be a proinflammatory mediator in several diseases (Sastre and Del Pozo, 2012). PGE_2_ was shown to induce Th2 cell development, Th2 cytokines secretion, and production of immunoglobulin E (Kaur et al., 1999). However, in respiratory diseases, PGE_2_ is important because of the multiplicity of its effects on immune response. PGE_2_ exerts a beneficial role in asthma via protection from lung smooth muscle proliferation and some anti-inflammatory effects (Sastre and Del Pozo, 2012). PGE_2_ may reduce mast cells activity and relax smooth muscle (Torres et al., 2015). In a clinical trial, PGE_2_ showed bronchodilatory effects via inhibition of inflammatory mediators (Chung, 2005).

The aim of this project was to compare the pharmacological activity of different NS preparations with a defined amount of TQ. This study investigated the activity of distinct NS preparations on asthma-related targets such as the release of IL-2, IL-6, and PGE_2_ from peripheral mononuclear human blood cells and a human epithelial cell line (A549) to provide the basis of a clinical study (Koshak et al., 2017b).

## Materials and Methods

### Initial Preparation of *Nigella sativa* Samples

Two main types of NS preparations were obtained. First, a series of laboratory prepared extracts from crude NS seeds (Bafart Company – Jeddah, Saudi Arabia). Second, commercial NS oil products. Whole NS seeds (10 g) were crushed and used for preparing each of six different NS preparations (Extracts 1–6, **Table 1**). One hundred milliliter of combined extraction solvent with different ratios (ethanol:water) were used to produce the extracts 1–5 by means of maceration over 72 h and extract 6 by means of decoction as following; extract no.1 (100:0), 2 (80:20), 3 (60:40), 4 (30:70), 5 (0:100), 6 (0:100 boiled for 30 min). After filtration, the ethanol was evaporated using a rota evaporator until constant volume, and then the remaining water was removed by freeze-drying. Single extraction was carried out for Extracts 1–6 and the extraction yields were as follows; extract no.1 (32%), 2 (12%), 3 (10%), 4 (12%), 5 (9%), 6 (15%). On the other hand, commercial ready-made NS oil products were obtained from different producers (extracts 7–10) as mentioned in **Table 1**. Then, all NS preparations were processed according to the analysis step (2.2).

**Table 1:** The list of obtained NS preparations and their thymoquinone concentration.

NS Preparation	Type of extract	Thymoquinone concentration (w/w) %
Extract 1	100% Ethanol	0.4%
Extract 2	80% Ethanol: 20% Water	0.01%
Extract 3	60% Ethanol: 40% Water	0.04%
Extract 4	30% Ethanol: 70% Water	0.005%
Extract 5	100% Water	<0.005%
Extract 6	100% Boiled water (30 min boiling)	Not detected
Extract 7	Super critical fluid (SCF) extract (Sami Labs Ltd., India)	2.4%
Extract 8	Cold pressed NS oil capsules (The Blessed Seeds, United Kingdom)	0.6%
Extract 9	Cold pressed NS oil capsules (Sanct Bernhard, Germany)	0.2%
Extract 10	Cold pressed NS oil capsules (Marnys S.A., Spain) (A licensed herbal product in Saudi Arabia)	0.7%


*Extracts 1 to 6 are laboratory prepared. Extracts 7–10 are ready-made commercial products.*

### Analysis of Thymoquinone Concentration Among *Nigella sativa* Samples

The HPLC method for the analysis of TQ was developed using the approach of (Hadad et al., 2012). The analysis was performed using a WATERS 2695 HPLC instrument attached to Waters 996 Photodiode Array Detector. (HPLC-PDA). The used HPLC column was phenomenex^®^ P/NO 00F-4252-E0, Desc Luna 5u C18(2) 100 Å, size 150 × 4.6 mm 5 μ, S/NO 312985-48.

All NS preparations were dissolved in 1 ml methanol in 1.5 ml Eppendorf tubes separately, vortexed for 10 s, and centrifuged for 2 min at 10,000 rpm to separate any remaining solid material. Afterwards, the supernatant was transferred to an HPLC amber vial via 0.45 mm filter for analysis with HPLC. Twenty microliter of each NS preparation were injected using linear gradient methanol–water (20–80 v/v, initial concentration) mobile phase over 10 min at gradient rate of 10%/min (80–20 v/v, final concentration) and with 12 min running time at 1.5 ml/min flow rate. TQ peak were detected in 10.3 min (retention time) at wavelength 254 nm. All samples were submitted in triplet and the average reading results were used. Then, TQ concentrations were determined in the samples using a calibration curve drawn from a series of known standard TQ concentrations.

Thymoquinone (purity > 98%) by Cayman Chemicals was used to prepare a range of serial dilutions of TQ for the calibration curve. The peak area of each standard TQ concentration (0.005, 0.05, 0.1, 0.2, 0.4, 0.6 mg/ml) detected at wavelength 254 nm was utilized to draw the calibration curve in Microsoft Excel. Then, the peak area of each NS preparation (in final prepared concentrations; extract 1 = 84, extract 2 = 36, extract 3 = 80, extract 4 = 109, extract 5 = 138, extract 6 = 125, extract 7 = 27, extract 8 = 64, extract 9 = 110, extract 10 = 65 mg/ml) were employed in the calibration curve, and the TQ concentrations were determined accordingly.

### Preparation of *Nigella sativa* Samples for the *in vitro* Analysis

Twenty milligram of each NS preparation was dissolved in 1ml dimethyl sulfoxide (DMSO, Merck Millipore) solvent inside a 1.5-ml Eppendorf tube using a rotamixer for 10 s. This was followed by centrifugation for 5 min at 1300 rpm for separation of insoluble substances. Then, the clear supernatant was re-diluted in DMSO to produce a final concentration of 10 or 100 μg/ml. Also, the standard TQ (Sigma) was prepared by using DMSO solvent to produce concentrations of 0.1, 1, 5, and 10 μM as required.

### Isolation of Human Peripheral Monocytes and T-Lymphocyte Cells for *in vitro* Analysis

Human blood from three different volunteer healthy donors, at the University Medical Centre Freiburg, was obtained in 450 ml buffy coats. The isolation of human monocytes and T-lymphocyte cells was carried out according to a standardized protocol (Noble and Cutts, 1968; English and Andersen, 1974). In six CELLSTAR^®^ (VWR) 50 ml centrifuge tubes, 25 ml of Lymphocyte Separation Medium (LSM 1077, PAA Laboratories GmbH) were added to each tube. Then, 20 ml of human blood were carefully added into each tube. These tubes were applied to centrifugation with Thermo Scientific^TM^ Megafuge^TM^ at 1800 rpm and acceleration 1 for 1 h at room temperature. After centrifugation, the phase containing mononuclear cells was transferred to 50 ml CELLSTAR^®^ tube prefilled with a 10 ml of Dulbecco’s phosphate buffered saline (DPBS, GIBCO^®^, Life Technologies) solution for washing. This was completed to 50 ml with the addition of more DPBS solution. Then, centrifugation for 10 min at 1600 rpm, normal acceleration and room temperature were performed. The supernatant was discarded, and DPBS was added on top of the buffy coat layer to fill it up to 50 ml. Afterwards, centrifugation at 1200 rpm for 10 min; the supernatant discarded, and 20 ml of DPBS was added with proper mixing. Additional DPBS was added to fill the tube up to 50 ml. The last step was repeated, and the supernatant was discarded, and the mononuclear cells remained in the pellet. Finally, the cells were re-suspended in 50 ml RPMI-1640 (Roswell Park Memorial Institute medium 1640, GE Healthcare) fortified with 10% human serum (PAA Coelbe). Cells were then seeded in the appropriate culture plates. An additional washing step after 1 h removed the T cells and enriches the monocytes.

### Monocytes and T-Lymphocyte Cells Stimulation

Twenty-four-well cell culture plate was seeded with 5 × 10^5^ cells/well resuspended in 1 ml culture medium (RPMI 1640 + 10% human serum) and incubated at 37°C for 1 h. Then the medium was changed, and the cells were further incubated for another 1 h at 37°C. Next, the NS preparations (10 and 100 μg/ml) and standard TQ (10 μM) were added to the respective wells. Generally, in each plate, two wells without extracts served as positive (cells + stimulant + DMSO) and negative (cells + DMSO) controls. After 30 min of incubation with the extracts, monocytes were stimulated with 10 ng/ml of LPS (from *Salmonella enterica* serotype typhimurium, Sigma-Aldrich) and T-lymphocytes were stimulated with 100 ng/ml staphylococcal enterotoxin B (SEB, Sigma-Aldrich). Cells were then incubated for 24 h in 5% CO_2_ at 37°C.

### Human Lung Epithelial Cells (A549) Stimulation

A549 cells (Sigma-Aldrich) were seeded (5 × 10^5^ cells/well) in 24-well plates and treated with 50 μl/well of each NS preparation (in concentrations of 10 and 100 μg/ml) and standard TQ (10 μM). In each plate, two wells without extracts served as a positive (cells + stimulant + DMSO) and negative (cells + DMSO) controls. After 30 min of incubation, 20 μl of the stimulant IL-1β (10 U/ml) (Roche Life Science) was added to all wells containing extracts and the positive control. All plates were further incubated for 24 h in 5% CO_2_ at 37°C before the supernatant was harvested for measurements.

### Enzyme-Linked Immunosorbent Assay for Interleukin-6, Interleukin-2, and Prostaglandin E2 Determination

After the end of the incubation period, supernatants were collected and centrifuged at 1000 *g* for 5 min at 4°C. PGE_2_ production were assessed in the supernatants with a commercially available enzyme immunoassay (EIA) kits (Biotrend, Cologne, Germany or Cayman Chemicals, Ann Arbor, Michigan, United States, respectively, and the cytokines IL-6, IL-2 by enzyme-linked immunosorbent assay (ELISA) kits according to the manufacturer’s instructions (Human IL-6 ELISA ready-SET-GO kit by eBioscience; Human IL-2 ELISA kit by R&D Systems). All experiments were replicated at least three times with different preparations of cells from different blood donors or cell line.

### Statistical Analysis

The results were statistically analyzed using one-way ANOVA with *post hoc* Dunnett’s test to investigate significant effects of each NS preparation against the control group. For each experiment, *P* value < 0.05 was considered statistically significant.

### Consent Procedure for Human Blood Samples

Buffy coats from healthy blood donors were obtained from the Institute of Cell and Gene therapy, University Hospital of Freiburg under approval from the local ethical committee (University of Freiburg).

## Results

### *Nigella sativa* Preparations and Their Thymoquinone Content

Ten different NS preparations, which varied in their TQ concentrations, were used in this study to compare their anti-inflammatory effects in primary human monocytes and T-lymphocytes as well as in lung epithelial cells (A549) (**Table 1**).

The levels of TQ in the NS preparations can be attributed to the extraction and preparation methods as well as the origins of the NS seeds. Among the commercial NS preparations (Extracts 7 to 10), extract 7 had the highest concentration of TQ, followed by extract 10. Among the laboratory prepared extracts (Extracts 1 to 6), extract 1 showed the highest level of TQ due to the solubility characteristics of TQ, which is highly soluble in organic solvents (**Table 1**).

### Effect of *Nigella sativa* Preparations on Inflammatory Mediators of Human T-Lymphocytes

**FIGURE 1:**
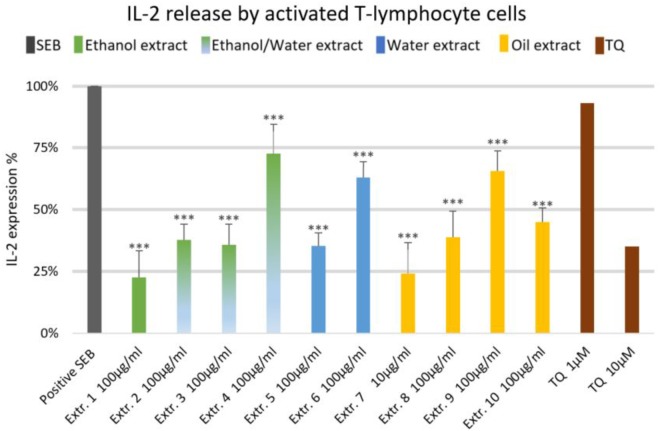
Effects of NS preparations on interleukin-2 production by SEB-induced T-lymphocyte cells (*n* = 3). Significant effects are indicated by an asterisk (^∗∗∗^*P* < 0.001).

**FIGURE 2:**
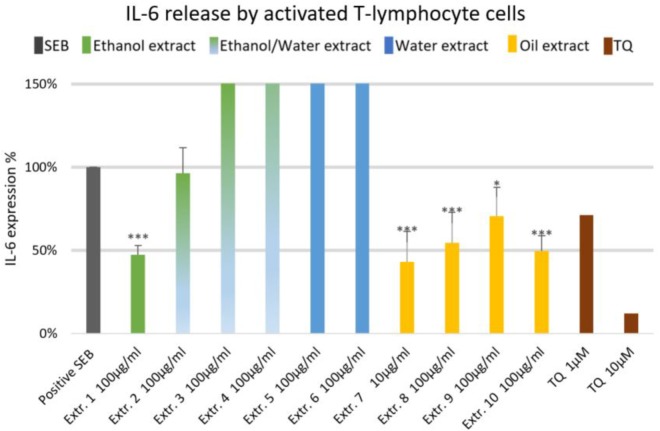
Effects of NS preparations on interleukin-6 production by SEB-induced T-lymphocyte cells (*n* = 3). Significant effects are indicated by asterisks (^∗^*P* < 0.05, ^∗∗∗^*P* < 0.001).

**FIGURE 3:**
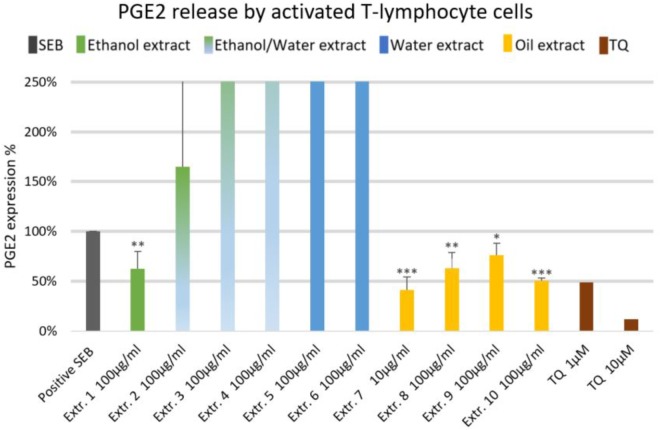
Effects of NS preparations on prostaglandin E2 production by SEB-induced T-lymphocyte cells (*n* = 3). Significant effects are indicated by asterisks (^∗^*P* < 0.05, ^∗∗^*P* < 0.01, ^∗∗∗^*P* < 0.001).

The effect of NS preparations on the release of the inflammatory mediators IL-2, IL-6, and PGE_2_ was investigated in SEB-induced human T-lymphocytes. All NS preparations suppressed SEB-induced IL-2 release from T-lymphocytes, which was significant (by more than 50%) with extracts 1, 2, 3, 5, 7, 8, and 10. TQ potently prevented SEB-induced IL-2 release with the dose of 10 μM (**Figure 1**). The ethanolic and oily extracts 1, 7, and 10 significantly reduced (by more than 50%) SEB-induced IL-6 release from T-lymphocyte cells (**Figure 2**). Conversely, SEB-induced IL-6 release from T-lymphocytes was further increased with water or mixed (water/ethanol) extracts 3, 4, 5, and 6. TQ potently prevented SEB-induced IL-2 release in the doses of 1 and 10 μM (**Figure 2**). SEB-induced PGE_2_ release from T-lymphocytes was significantly inhibited (by more than 50%) with the oily extracts 7 and 10 (**Figure 3**). Conversely, there was a significant enhancement of PGE_2_ release (by more than 150%) with the mixed (water/ethanol) extracts, and particularly with pure water extracts (by up to 10 times). TQ potently prevented SEB-induced PGE_2_ release in the doses of 1 and 10 μM (**Figure 3**).

### Effect of *Nigella sativa* Preparations on Inflammatory Mediators in Human Monocytes

**FIGURE 4:**
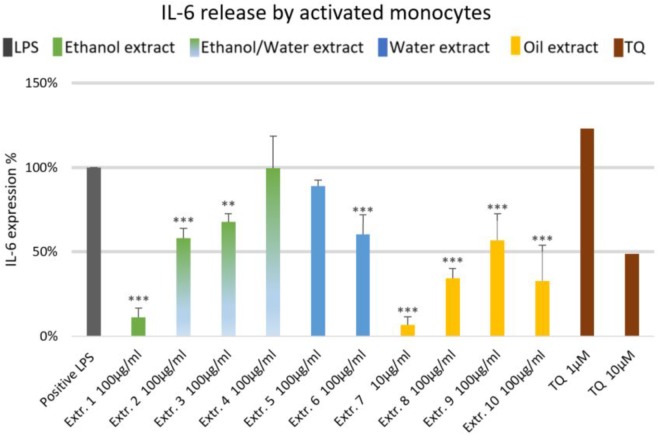
Effects of NS preparations on interleukin-6 production in LPS-induced monocytes (*n* = 3). Significant effects are indicated by asterisks (^∗∗^*P* < 0.01, ^∗∗∗^*P* < 0.001).

**FIGURE 5:**
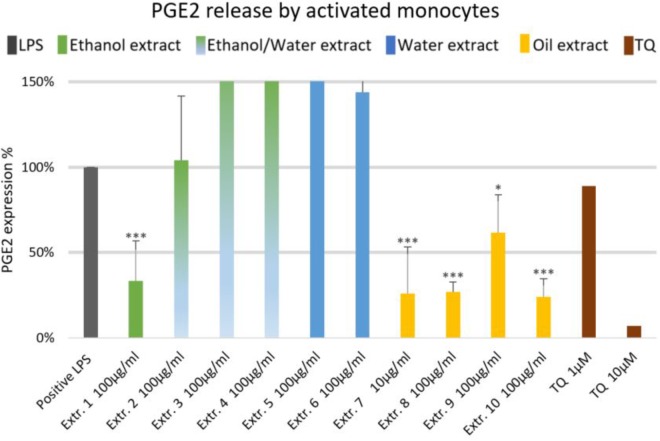
Effects of NS preparations on prostaglandin E2 release by LPS-induced monocytes (*n* = 3). Significant effects are indicated by asterisks (^∗^*P* < 0.05, ^∗∗∗^*P* < 0.001).

The effects of NS preparations on the release of inflammatory mediators IL-6 and PGE_2_ was investigated in LPS-induced monocytes. A suppression of LPS-induced IL-6 release in human monocytes was observed with most extracts, and a significant inhibition occurred (by more than 50%) with the ethanolic and oily extracts 1, 7, 8, and 10 (**Figure 4**). Additionally, LPS-induced PGE_2_ release was significantly suppressed in monocytes (by more than 50%) with the ethanolic and oily extracts 1, 7, 8, and 10 (**Figure 5**). TQ potently prevented LPS-induced IL-6 and PGE_2_ release in the doses of 10 μM (**Figures 4, 5** right columns).

### Effect of *Nigella sativa* Preparations on Inflammatory Mediators of A549 Human Lung Epithelial Cells

**FIGURE 6:**
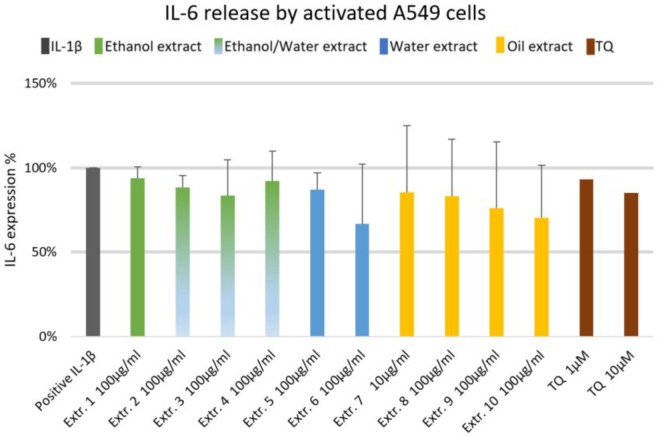
Effect of NS preparations on interleukin-6 production by interleukin-1β-induced A549 cells (*n* = 3). There was no significant effects.

**FIGURE 7:**
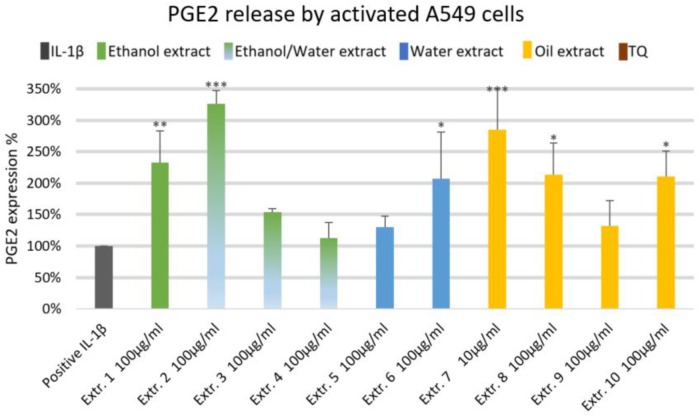
Effect of NS preparations on prostaglandin E2 production by interleukin-1β-induced A549 cells (*n* = 3). Significant effects are indicated by asterisks (^∗^*P* < 0.05, ^∗∗^*P* < 0.01, ^∗∗∗^*P* < 0.001).

We did not observe significant inhibition of IL-1β-induced IL-6 release by any one of the NS preparations and TQ in A549 lung epithelial cells (**Figure 6**). However, extracts 6 and 7 showed a slight tendency to inhibit IL-6 release in IL-1β-induced A549 lung epithelial cells. Conversely, the release of IL-1β-induced PGE_2_ was markedly (by more than 150%) increased with extracts 1, 2, 6, 7, 8, and 10. More interestingly, the release of IL-1β-induced PGE_2_ with TQ was over the detection limit (**Figure 7**).

### Summary of the Effects of *Nigella sativa* Preparations on the Release of Inflammatory Mediators in Pre-clinical *in vitro* Models

The oily extracts 7 (a supercritical fluid extract) and 10 (a commercial oil product by Marnys S.A., Spain), which are characterized by the highest level of TQ, showed the most remarkable effect on suppressing inflammatory mediators IL-2, IL-6, and PGE_2_ released from human T-lymphocytes. In addition, these two extracts showed a marked suppression of IL-6 and PGE_2_ release in human monocytes. The extracts 1, 2, and 8 also revealed a suppression of inflammatory mediators in T-lymphocyte and monocytes but in a limited number of mediators as compared to extracts 7 and 10, which showed a much broader range of activities. On the other hand, the extracts 1, 2, 7, 8, 10 had a remarkable effect enhancing the release of PGE_2_ from A549 human lung epithelial cells (**Table 2**). Therefore, the oily extracts 7 and 10 showed the most potent and favorable effects in the context of asthma, due to their anti-inflammatory actions by suppressing the release of inflammatory mediators in human immune cells (T-lymphocyte and monocyte cells) as well as their possible bronchodilatory effect by enhancement of PGE_2_ release in A549 human lung epithelial cells.

**Table 2:** Summary of the most active NS preparations by more than 50% suppression or 150% enhancement of inflammatory mediators in cellular *in vitro* models of inflammation (*n* = 3).

Sample	T-Lymphocyte Cells			Monocytes		A549
			
	IL-6	IL-2	PGE_2_	IL-6	PGE_2_	PGE_2_
Extract 1	↓≥50%	↓≥50%		↓≥50%	↓≥50%	↑≥150%
Extract 2		↓≥50%				↑≥150%
Extract 7	↓≥50%	↓≥50%	↓≥50%	↓≥50%	↓≥50%	↑≥150%
Extract 8		↓≥50%		↓≥50%	↓≥50%	↑≥150%
Extract 10	↓≥50%	↓≥50%	↓≥50%	↓≥50%	↓≥50%	↑≥150%


## Discussion

The effects of NS preparations on the inhibition of inflammatory mediators (IL-6, IL-2, PGE_2_) were investigated in human primary T-lymphocyte cells, human primary monocytes, and A549 lung epithelial cells. We show here that TQ-rich extracts (especially extract no. 7 and 10) appeared to be the most potent extracts in the context of anti-inflammatory/immunomodulatory activity. The variability of the *in vitro* activity of NS preparations correlates with differences in the chemical composition, especially the TQ content, of the NS preparations. Furthermore, our results showed that NS oil inhibited the release of some cytokines with inflammatory properties, which are found to be upregulated in patients with asthma. Therefore, NS may have a corticosteroid-like effect in abolishing the upregulation of some inflammatory mediators to induce remission of asthmatic symptoms. Interestingly, NS also enhanced the release of PGE_2_, which are thought to have a bronchodilatory effect in bronchial epithelium. This study enabled us to identify the most active NS preparations in various *in vitro* models of Asthma.

In human T-lymphocytes, the suppressive effect of NS on IL-2, IL-6, and PGE_2_ release is not reported in the literature so far. However, there was a suppression of serum IL-2 in mice treated with NS fixed oil (Abbas et al., 2005). This suppressive effect on IL-2 release from T lymphocyte cells may be beneficial in the context of asthma as it is considered a pro-inflammatory mediator and found in a high level in BALF of patients with asthma. The suppressive effects of NS on IL-6 release from SEB-treated T lymphocytes may also be beneficial in the context of asthma as IL-6 is considered as a pro-inflammatory mediator and found at high levels in the serum of patients with asthma. Also, the administration of NS volatile oil significantly lowered the plasma level of PGE_2_ in rats (Salim, 2010). In *in vitro* investigations, TQ caused a general anti-inflammatory activity via inhibition of COX-1 and COX-2, which subsequently blocked PGE_2_ production (Marsik et al., 2005). Carvacrol, a volatile oil constituent of NS, inhibited the release of PGE_2_ catalyzed by COX-2 (Landa et al., 2009a). This suppressive effect of PGE_2_ release from T-lymphocytes may be considered a favorable effect due to the general pro-inflammatory properties of PGE_2_ and the enhancement effect of PGE_2_ on Th2 and IgE responses. Interestingly, there was a positive relationship between the TQ level of the oily NS preparations and the inhibition of the inflammatory mediators IL-2, IL-6, and PGE_2_.

In human monocytes, the anti-inflammatory effects observed by the treatment with NS are not reported in the literature so far. Some studies showed that TQ alone suppressed the expression of IL-6 from *Mycobacterium tuberculosis* infected human monocyte THP-1 cells (Mahmud et al., 2017). Furthermore, TQ inhibited the production of IL-6 in LPS-activated murine macrophage-like RAW264.7 cells (Hossen et al., 2017). These data are in line with the data we obtained by TQ in LPS-induced human monocytes. The suppressive effect of IL-6 release stimulated by LPS from monocytes might be beneficial in the context of asthma as it is considered a pro-inflammatory mediator and found in high serum levels of patients with asthma. The inhibitory effect of NS on LPS-induced PGE_2_ release in monocytes may be considered as a favorable effect due to the general systemic pro-inflammatory properties of PGE_2_ and the enhancement effect of PGE_2_ on Th2 and IgE responses.

We also present novel data on the effects of NS in A549 human lung epithelial cells, as we demonstrated an enhanced release of IL-1-induced PGE_2_, but with no significant effect on IL-6 synthesis. This is not in line with other studies in lung tissues and cells. A previous study demonstrated that NS fixed oil significantly suppressed mRNA IL-6 in whole lung tissue of rats (Shahzad et al., 2009). Moreover, TQ suppressed the expression of IL-6 from *M. tuberculosis* infected A549 cells (Mahmud et al., 2017). Inhibitory effects of TQ were also shown for IL-6 expression in human proximal tubular epithelial cells stimulated with advanced glycation end products (Sayed and Morcos, 2007). Suppression of IL-6 may possess beneficial anti-inflammatory effects in bronchial epithelial cells of asthmatic patients (Mattoli et al., 1992). Unlike beta-agonists (a conventional treatment in asthma), which can enhance IL-6 expression in the lung, NS had no, or only mild inhibitory effects on IL-1-induced IL-6 release (Edwards et al., 2007). The stimulatory effect of NS on PGE_2_ release from A549 human lung epithelial cells has not been reported so far. However, increased PGE_2_ release was shown in perfused guinea-pig lung preparation using NS oil (Saleh et al., 2012). Interestingly, the increased PGE_2_ release by TQ-rich NS preparations may have a favorable effect in the context of asthma as several studies showed that PGE_2_ may possess beneficial local protective and bronchodilatory effects in the airways of the lung (Chung, 2005; Sastre and Del Pozo, 2012; Torres et al., 2015).

This *in vitro* study served as a basis for developing the clinical study of NS oil in patients with asthma conducted by our group (Koshak et al., 2017b). Consequently, the mediators investigated here were general inflammatory targets associated with asthma. In addition, it would be of interest to investigate other classical targets in asthma disease and to compare the effects of NS preparations on the release of Th2 cytokines IL-4, IL-5, and IL-13 in *in vitro* models.

## Conclusion

The results of this work showed variability in the anti-inflammatory/immunomodulatory effects of different NS preparations. Direct *in vitro* evidence points to the release of some asthma-related cytokines and a prostanoid with inflammatory properties (IL-2, IL-6, PGE_2_) from human immune cells (including T-lymphocytes and monocytes). These were potently modulated by NS preparations rich in fatty oils. There was a positive relationship between the levels of TQ and the inhibition of inflammatory mediators in T-lymphocytes and monocytes. Interestingly, the oily preparations showed an increase in PGE_2_ production in lung epithelial cells, which may possess a favorable effect in the context of asthma. Ultimately, the oily TQ-rich NS preparations appeared to be the most potent preparations and are recommended for further investigations in clinical trials.

More broadly, this method of pharmacological screening combined with a chemical analysis of single or more active compounds should be implemented for other herbal products to identify most active chemically characterized preparations. Such chemical analysis of active compounds is essential prior to using plant extracts in clinical studies. This is of a particular importance for many herbal medicines currently under clinical and preclinical investigation in the fast-developing economies of the global South. Also, further investigations are suggested on the effect of NS on other inflammatory mediators as well as the mechanism by which these mediators are inhibited.

## Data Availability

The raw data supporting the conclusions of this manuscript will be made available by the authors, without undue reservation, to any qualified researcher.

## Author Contributions

MH and EK encouraged AK to investigate the immunomodulatory activity of different NS preparations and supervised the findings of this work and directed the project. BF helped supervise the project and verified the analytical methods. AK, NY, and BF conceived and planned the experiments. AK prepared and characterized the experimental samples, designed the figures and performed the statistical calculations, and wrote the manuscript with input from all authors. AK and NY carried out the experiment and analyzed the data in consultation with BF. BF and EK contributed to the interpretation of the results. All authors provided critical feedback and helped shape the research, analysis, and manuscript.

## Conflict of Interest Statement

BF is employed by VivaCell Biotechnology, Germany. The remaining authors declare that the research was conducted in the absence of any commercial or financial relationships that could be construed as a potential conflict of interest.

## Abbreviations

DMSO: dimethyl sulfoxide
DPBS: Dulbecco‘s phosphate buffered saline
HPLC: high-performance-liquid chromatography
IL: interleukin
LPS: lipopolysaccharides
NS: *Nigella sativa* L.
PGE_2_: prostaglandin E_2_
SEB: *Staphylococcal enterotoxin* B
TQ: thymoquinone
